# Establishment and validation of a nomogram to select patients with metastatic sarcomatoid renal cell carcinoma suitable for cytoreductive radical nephrectomy

**DOI:** 10.3389/fonc.2023.1239405

**Published:** 2023-10-23

**Authors:** Yulin Zhou, Yufeng Gu, Chaopeng Tang, Jie Dong, Song Xu, Zhengcheng Sheng, Xiaodong Zhao, Jun Hu, Tianyi Shen, Haowei He, Xiaoming Yi, Wenquan Zhou, Le Qu, Jingping Ge, Conghui Han

**Affiliations:** ^1^ Medical College of Soochow University, Suzhou, China; ^2^ Department of Urology, Affiliated Jinling Hospital, Medical School of Nanjing University, Nanjing, China; ^3^ Department of Urology, Xuzhou Central Hospital, Xuzhou, China

**Keywords:** metastatic, sarcomatoid renal cell carcinoma, cytoreductive radical nephrectomy, nomogram, prognosis, SEER database

## Abstract

**Introduction:**

Metastatic renal cell carcinoma (mRCC) with sarcomatoid features has a poor prognosis. Cytoreductive radical nephrectomy (CRN) can improve prognosis, but patient selection is unclear. This study aimed to develop a prediction model for selecting patients suitable for CRN.

**Materials and methods:**

Patients with a diagnosis of mRCC with sarcomatoid features in the Surveillance, Epidemiology, and End Results (SEER) database between 2010 and 2015 were retrospectively reviewed. CRN benefit was defined as a survival time longer than the median overall survival (OS) in patients who did not receive CRN. A prediction nomogram was established and validated using the SEER cohort (training and internal validation) and an external validation cohort.

**Results:**

Of 900 patients with sarcomatoid mRCC, 608 (67.6%) underwent CRN. OS was longer in the CRN group than in the non-CRN group (8 vs. 6 months, hazard ratio (HR) = 0.767, *p* = 0.0085). In the matched CRN group, 124 (57.7%) patients survived >6 months after the surgery and were considered to benefit from CRN. Age, T-stage, systematic therapy, metastatic site, and lymph nodes were identified as independent factors influencing OS after CRN, which were included in the prediction nomogram. The monogram performed well on the training set (area under the receiver operating characteristic (AUC) curve = 0.766, 95% confidence interval (CI): 0.687–0.845), internal validation set (AUC = 0.796, 95% CI: 0.684–0.908), and external validation set (AUC = 0.911, 95% CI: 0.831–0.991).

**Conclusions:**

A nomogram was constructed and validated with good accuracy for selecting patients with sarcomatoid mRCC suitable for CRN.

## 1 Introduction

Renal cell carcinoma (RCC) is the most prevalent type of malignant tumor of the kidney, accounting for 21.82% of urinary tract tumors; its prevalence is only second to that of bladder cancer and prostate cancer in the urinary tract (1). The incidence of RCC is increasing annually (2); approximately 17% of patients with RCC present with metastatic disease at the first visit (3). CARMENA, a limited classical randomized controlled trial, demonstrated that cytoreductive nephrectomy followed by sunitinib was not superior to sunitinib alone in some patients with metastatic RCC (mRCC) (4), suggesting that not all mRCC cases are suitable for cytoreductive nephrectomy.

RCC with sarcomatoid features, also known as sarcomatoid RCC, was first discovered and described by Farrow et al. (5). After further careful scrutiny, in 2004, the World Health Organization proposed that sarcomatoid RCC is no longer considered a separate pathological subtype of RCC (6). In 2012, the International Society of Urological Pathology suggested that sarcomatoid RCC should be classified as World Health Organization/International Society of Urological Pathology grade 4 and revealed that its biological behavior is more aggressive than that of RCC (7). Although sarcomatoid differentiation has been observed in only 5% of RCC cases, this type of tumor accounts for approximately 20% of advanced renal cancer cases (8). RCC with sarcomatoid features is associated with a poorer prognosis and shorter survival (9).

However, no therapeutic strategies are effective for mRCC with sarcomatoid features. Current guidelines only recommend cytoreductive nephrectomy for patients with mRCC with favorable and intermediate risk levels defined by the International Metastatic Renal Cell Carcinoma Database Consortium (10). However, owing to the heterogeneity among kidney cancers, the efficacy of cytoreductive nephrectomy varies greatly in patients with sarcomatoid mRCC.

Cytoreductive nephrectomy includes two surgical approaches, namely cytoreductive partial nephrectomy and cytoreductive radical nephrectomy (CRN). Among patients with mRCC who underwent cytoreductive nephrectomy, only 2%–4% of them underwent cytoreductive partial nephrectomy (11, 12). Therefore, this study exclusively focused on CRN for mRCC with sarcomatoid features.

This study aimed to construct a nomogram for selecting patients with sarcomatoid mRCC who can benefit from CRN using data from the Surveillance, Epidemiology, and End Results (SEER) database and our multicenter follow-up cohort.

## 2 Materials and methods

### 2.1 Patients and data sources

SEER*Stat software (version 8.4.0.1) was used to extract data from patients with sarcomatoid mRCC who were diagnosed between 2010 and 2015. For this study, we signed a data agreement and used the SEER database under the username 17749-Nov2020. Because SEER is a publicly accessible database containing deidentified data, an ethical review is not needed. Sarcomatoid mRCC was defined as kidney parenchyma with sarcomatoid features and distant metastases (American Joint Committee on Cancer 7th M1). The histology recoded broad groupings: 8,140–8,389 adenomas and adenocarcinomas were included according to the 2016 WHO histopathological classification of renal cell tumors.

For external validation (*n* = 55), a retrospective cohort of patients with sarcomatoid mRCC who underwent CRN between January 2010 and December 2020 was extracted from the medical records of three medical centers in China: Nanjing University Jinling Hospital, Affiliated Hospital of Medical School, Nanjing University; Changhai Hospital, Naval Medical University; and Qilu Hospital of Shandong University. Ethical approval was obtained from the ethics committee of each institution. In cases in which individual patient consent was not required, the chairperson of the ethics committee waived the need for patient consent.

Patients aged ≥18 years and diagnosed with metastatic mRCC and sarcomatoid features were included. Patients with tumors without sarcomatoid features, unknown TNM stage, unknown survival data, nonunilateral tumor or unknown tumor laterality, not RCC, or unknown metastatic status (including bone, brain, liver, and lung) were excluded.

**Figure 1:**
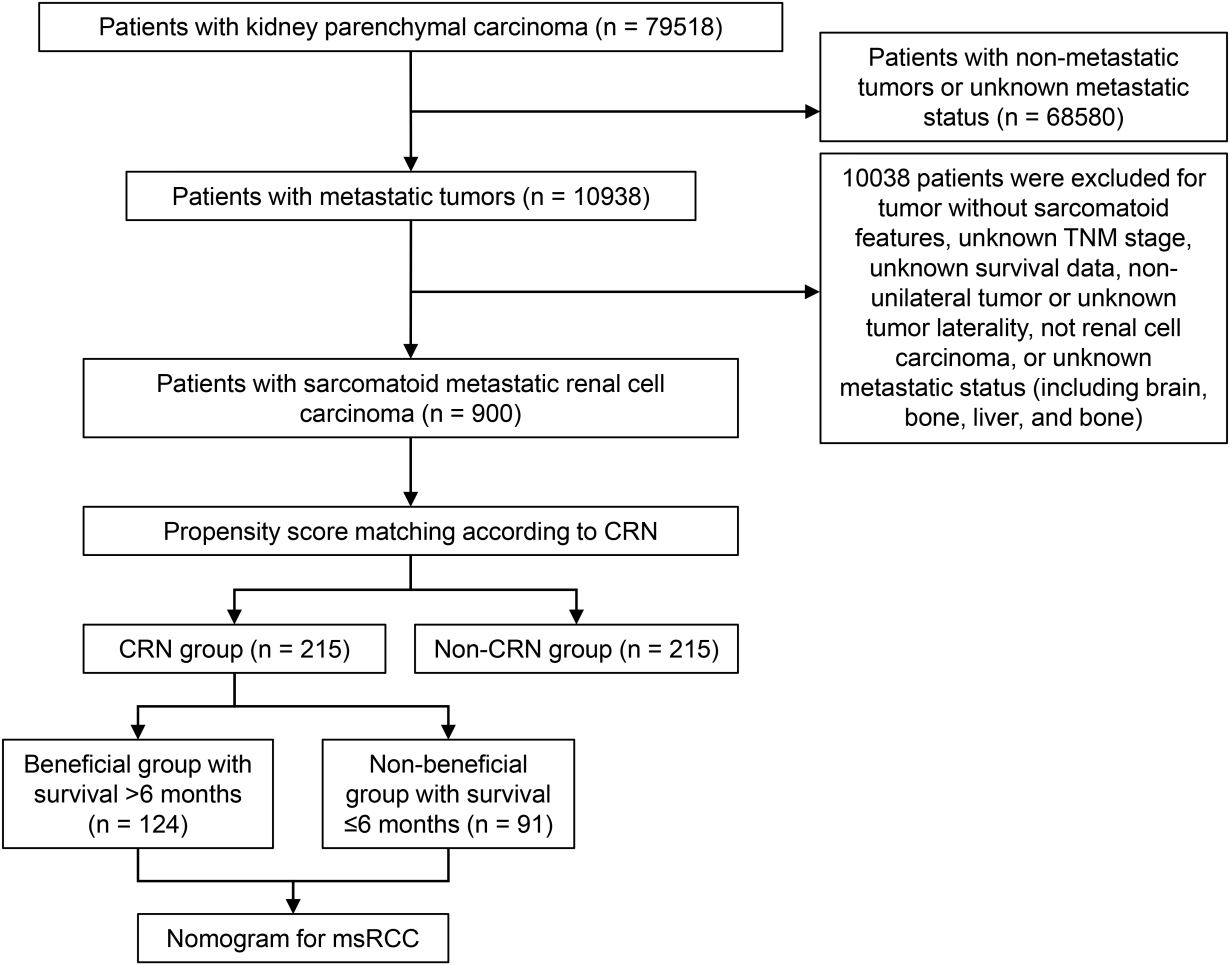
Flowchart of patient selection. CRN, cytoreductive radical nephrectomy; msRCC, metastatic sarcomatoid renal cell carcinoma.

CRN was defined as radical nephrectomy (RX Summ-Surg Prim Site (1998+) surgery code: 50). The screening process of the study population and the establishment of the predictive model is depicted in a flowchart (**Figure 1**).

### 2.2 Statistical analysis

According to whether the patients underwent CRN, the study population from the SEER database was divided into the CRN group and the non-CRN group. Multivariate logistic regression models using the stepwise method were used to predict CRN candidates to prove the necessity of propensity score matching (PSM). Multivariate Cox regression by the stepwise method was employed to determine the independent risk factors that would be included in the nomogram.

To minimize selection bias and baseline imbalance, PSM was used to match the non-CRN group to the CRN group. By logistic regression, a PS was generated using variables that could potentially influence treatment outcomes, such as age, sex, laterality, T-stage, N-stage, bone metastasis, brain metastasis, liver metastasis, lung metastasis, systematic therapy, metastatic site, and lymph nodes. The CRN and non-CRN groups were 1:1 matched using the nearest PS on the logit scale with a caliper of 0.08 without replacement. The balance of covariables in both groups before and after matching was evaluated by standardized differences, and a standardized difference of <10% was considered to indicate sufficient balance.

The Chi-square test was used to determine the significance of the difference in categorical variables. Cox proportional hazard regression was used to determine independent prognostic factors. Hazard ratios (HRs) were calculated with a 95% confidence interval (CI). Statistical analysis was performed with R version 4.2.1 (http://www.r-project.org/). A two-sided *p*-value of <0.05 was considered statistically significant.

### 2.3 Establishment and validation of the nomogram

The median overall survival (OS) in the non-CRN group was 6 months. After PSM, patients with an OS of >6 months in the CRN group were classified as the “CRN benefit group.” Conversely, those with an OS of <6 months in the CRN group were classified as the “no CRN benefit group.”

To identify patients with sarcomatoid mRCC who may benefit from CRN, a prediction model was established using multivariate logistic regression analysis. The matched CRN group (*n* = 215) was randomly divided into two groups for training (*n* = 150) and internal validation (*n* = 65) with a ratio of 7:3. The logistic regression model comprised the independent predictor variables from multivariate Cox regression analysis, including age, T-stage, systemic therapy, metastatic site, and lymph nodes. This prediction model was built on the training set and displayed as a nomogram. The probability of patients with sarcomatoid mRCC benefiting from CRN was calculated by summing the scores for each selected variable. The area under the receiver operating characteristic (AUC) curve was used to determine the prediction efficiency (sensitivity and specificity) in the training, internal validation, and external validation cohorts.

To compare the predicted and observed outcomes, a calibration plot and decision curve analysis were performed, with a *p*-value of >0.05 indicating a good model fit. A classification system based on the nomogram was established to predict the probability that a patient with sarcomatoid mRCC may benefit from CRN. A predicted probability of >0.5 indicated a good chance of CRN benefit and hence longer survival; conversely, a predicted probability of ≤0.5 indicated that the patient may not benefit from CRN. The Kaplan–Meier method was performed to estimate OS and cancer-specific survival (CSS) in CRN benefit and no CRN benefit groups. This method was performed to verify that the model can identify patients who may benefit from CRN and subsequently evaluate the clinical utility of the model.

## 3 Results

### 3.1 Patient baseline characteristics

A total of 900 sarcomatoid mRCC cases were included in this study based on the SEER database using the following SEER variables: “primary site—labeled: C64.9—kidney, NOS,” and “Histology recoded broad groupings: 8,140–8,389 adenomas and adenocarcinomas.” Multivariate logistic regression (stepwise method) of all patients showed that the independent predictors of CRN recipients were T-stage, bone metastasis, liver metastasis, systematic therapy, and lymph nodes (**Table 1**).

**Table 1:** Multivariable logistic regression models (stepwise method) predicting the probability of cytoreductive radical nephrectomy recipients.

Variable	Odds ratio (95% confidence interval)	*p*-value
T-stage		
T1	Reference	
T2	2.14 (1.15–4.03)	0.017
T3	6.19 (3.73–10.4)	<0.001
T4	1.97 (1.12–3.50)	0.02
Bone metastasis		
No	Reference	
Yes	0.649 (0.462–0.912)	0.012
Liver metastasis		
No	Reference	
Yes	0.544 (0.364–0.815)	0.003
Systemic therapy		
None/Unknown	Reference	
Yes	3.42 (2.49–4.72)	<0.001
Lymph nodes		
None	Reference	
LN metastasis, NOS	0.378 (0.173–0.825)	0.014
SLN metastasis	0.73 (0.474–1.131)	0.155
MLN metastases	0.732 (0.488–1.103)	0.134

LN, lymph node; SLN, single lymph node; MLN, multiple lymph nodes.

Before matching, significant differences in T-stage, bone metastasis, liver metastasis, systematic therapy, and lymph nodes were found between the CRN and non-CRN groups, which were well balanced after matching (all *p* > 0.05), and 215 matched pairs of patients with metastatic sarcomatoid renal cell carcinoma with or without CRN were generated and enrolled in the subsequent analysis (**Table 2**). The baseline characteristics of the three cohorts (training, internal validation, and external validation cohorts) are shown in **Table 3**.

**Table 2:** Patient baseline characteristics before and after PSM (*n* (%)).

	Before PSM			After PSM		
	Non-CRN *N=292*	CRN *N=608*	P-value	Non-CRN *N=215*	CRN *N=215*	P-value
Age			0.856			0.118
<65	178 (61.0%)	376 (61.8%)		133 (61.9%)	116 (54.0%)	
≥65	114 (39.0%)	232 (38.2%)		82 (38.1%)	99 (46.0%)	
Sex			0.625			0.828
Female	86 (29.5%)	168 (27.6%)		59 (27.4%)	56 (26.0%)	
Male	206 (70.5%)	440 (72.4%)		156 (72.6%)	159 (74.0%)	
Laterality			1.000			0.562
Left	147 (50.3%)	306 (50.3%)		105 (48.8%)	98 (45.6%)	
Right	145 (49.7%)	302 (49.7%)		110 (51.2%)	117 (54.4%)	
T-stage			<0.001			0.695
T1	62 (21.2%)	34 (5.6%)		23 (10.7%)	30 (14.0%)	
T2	43 (14.7%)	52 (8.6%)		32 (14.9%)	34 (15.8%)	
T3	105 (36.0%)	420 (69.1%)		101 (47.0%)	99 (46.0%)	
T4	82 (28.1%)	102 (16.8%)		59 (27.4%)	52 (24.2%)	
N-stage			0.020			0.620
N0	161 (55.1%)	386 (63.5%)		130 (60.5%)	136 (63.3%)	
N1	131 (44.9%)	222 (36.5%)		85 (39.5%)	79 (36.7%)	
Bone metastasis:			0.001			0.538
No	171 (58.6%)	428 (70.4%)		141 (65.6%)	148 (68.8%)	
Yes	121 (41.4%)	180 (29.6%)		74 (34.4%)	67 (31.2%)	
Brain metastasis:			0.199			0.248
No	259 (88.7%)	557 (91.6%)		192 (89.3%)	183 (85.1%)	
Yes	33 (11.3%)	51 (8.4%)		23 (10.7%)	32 (14.9%)	
Liver metastasis			<0.001			0.702
No	219 (75.0%)	530 (87.2%)		180 (83.7%)	176 (81.9%)	
Yes	73 (25.0%)	78 (12.8%)		35 (16.3%)	39 (18.1%)	
Lung metastasis			1.000			0.283
No	109 (37.3%)	227 (37.3%)		85 (39.5%)	97 (45.1%)	
Yes	183 (62.7%)	381 (62.7%)		130 (60.5%)	118 (54.9%)	
Systematic therapy			<0.001			0.333
None/Unknown	196 (67.1%)	215 (35.4%)		122 (56.7%)	111 (51.6%)	
Yes	96 (32.9%)	393 (64.6%)		93 (43.3%)	104 (48.4%)	
Metastatic site:			<0.001			0.472
Other metastasis	27 (9.2%)	92 (15.1%)		26 (12.1%)	34 (15.8%)	
One of bone liver brain lung	159 (54.5%)	368 (60.5%)		130 (60.5%)	120 (55.8%)	
Multiple	106 (36.3%)	148 (24.3%)		59 (27.4%)	61 (28.4%)	
Lymph nodes			0.018			0.682
None	161 (55.1%)	386 (63.5%)		130 (60.5%)	136 (63.3%)	
LN metastasis, NOS	18 (6.2%)	16 (2.6%)		13 (6.0%)	8 (3.7%)	
SLN metastasis	52 (17.8%)	92 (15.1%)		32 (14.9%)	34 (15.8%)	
MLNs metastases	61 (20.9%)	114 (18.8%)		40 (18.6%)	37 (17.2%)	

**Table 3:** General characteristics of the training, internal validation, and external validation cohorts (*n* (%)).

	Training *N=150*	Internal validation *N=65*	External validation *N=55*	P-value
Age				0.293
<65	82 (54.7%)	34 (52.3%)	36 (65.5%)	
≥65	68 (45.3%)	31 (47.7%)	19 (34.5%)	
Sex				0.921
Female	38 (25.3%)	18 (27.7%)	15 (27.3%)	
Male	112 (74.7%)	47 (72.3%)	40 (72.7%)	
Laterality:				0.274
Left	64 (42.7%)	34 (52.3%)	29 (52.7%)	
Right	86 (57.3%)	31 (47.7%)	26 (47.3%)	
T-stage				0.005
T1	26 (17.3%)	4 (6.2%)	10 (18.2%)	
T2	18 (12.0%)	16 (24.6%)	18 (32.7%)	
T3	71 (47.3%)	28 (43.1%)	15 (27.3%)	
T4	35 (23.3%)	17 (26.2%)	12 (21.8%)	
N-stage				0.001
N0	94 (62.7%)	42 (64.6%)	19 (34.5%)	
N1	56 (37.3%)	23 (35.4%)	36 (65.5%)	
Bone metastasis:				0.018
No	101 (67.3%)	47 (72.3%)	48 (87.3%)	
Yes	49 (32.7%)	18 (27.7%)	7 (12.7%)	
Brain metastasis:				0.330
No	128 (85.3%)	55 (84.6%)	51 (92.7%)	
Yes	22 (14.7%)	10 (15.4%)	4 (7.3%)	
Liver metastasis:				0.014
No	120 (80.0%)	56 (86.2%)	53 (96.4%)	
Yes	30 (20.0%)	9 (13.8%)	2 (3.6%)	
Lung metastasis:				0.771
No	70 (46.7%)	27 (41.5%)	24 (43.6%)	
Yes	80 (53.3%)	38 (58.5%)	31 (56.4%)	
Systematic therapy:				0.439
None/Unknown	75 (50.0%)	36 (55.4%)	24 (43.6%)	
Yes	75 (50.0%)	29 (44.6%)	31 (56.4%)	
Metastatic site:				0.026
Other metastasis	23 (15.3%)	11 (16.9%)	18 (32.7%)	
One of bone liver brain lung	83 (55.3%)	37 (56.9%)	30 (54.5%)	
Multiple	44 (29.3%)	17 (26.2%)	7 (12.7%)	
Lymph nodes				<0.001
None	94 (62.7%)	42 (64.6%)	19 (34.5%)	
LN metastasis, NOS	4 (2.7%)	4 (6.2%)	17 (30.9%)	
SLN metastasis	26 (17.3%)	8 (12.3%)	11 (20.0%)	
MLNs metastases	26 (17.3%)	11 (16.9%)	8 (14.5%)	

**Figure 2:**
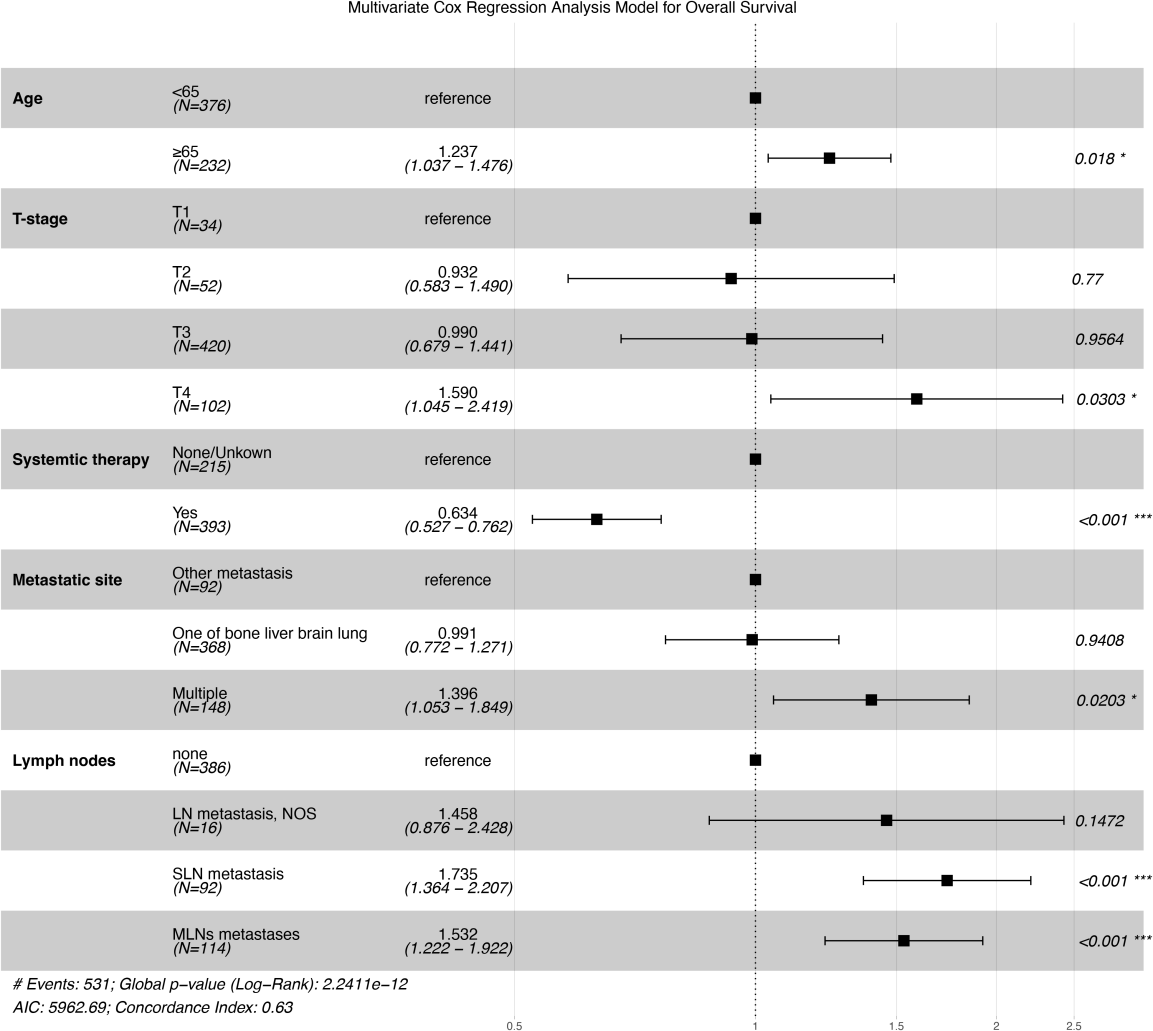
Forest plot of the results of multivariate Cox regression analysis. *p < 0.05, ***p < 0.001

In the nomogram, the independent prognostic predictive variables from the multivariate Cox regression (stepwise method) for OS included age, T-stage, systematic therapy, metastatic site, and lymph nodes (**Figure 2**).

### 3.2 Survival results

**Figure 3:**
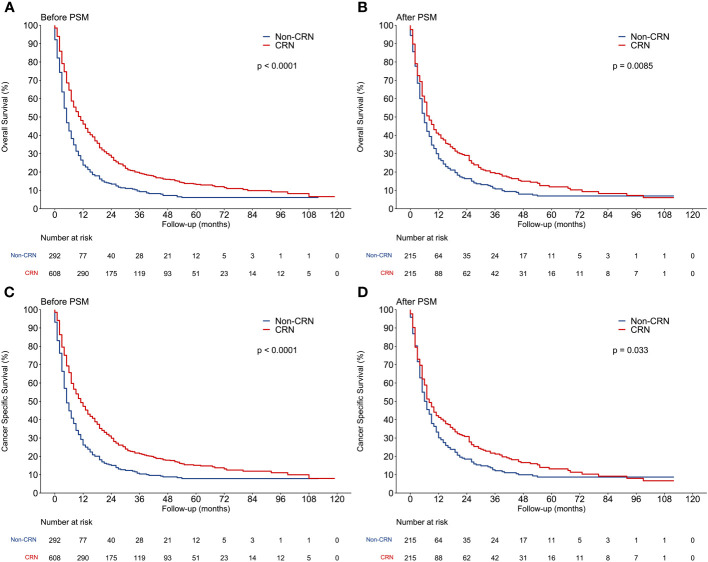
Comparison of overall survival and cancer-specific survival between CRN and non-CRN groups. **(A, C)** OS and CSS of the 2 groups before PSM. **(B, D)** OS and CSS of the 2 groups after PSM.

Either before or after PSM, Kaplan–Meier analysis revealed that the CRN group was associated with significantly better OS and CSS than the non-CRN group (**Figure 3**). Before PSM, the median OS and CSS were both 11 months in the CRN group and both 5 months in the non-CRN group. After PSM, the CRN group showed longer median OS (8 vs. 6 months, HR = 0.767, *p* = 0.0085) and longer median CSS (8 vs. 7 months, HR = 0.801, *p* = 0.033) than the non-CRN group.

### 3.3 Construction of a nomogram to identify candidates benefiting from CRN

After PSM, patients in the CRN group (*n* = 215) whose OS exceeded the median OS of the non-CRN group were assigned to the “CRN benefit group” (OS of >6 months, *n* = 124, 57.7%). The remaining patients were assigned to the “no CRN benefit group” (survival time of ≤6 months, *n* = 91, 42.3%).

**Figure 4:**
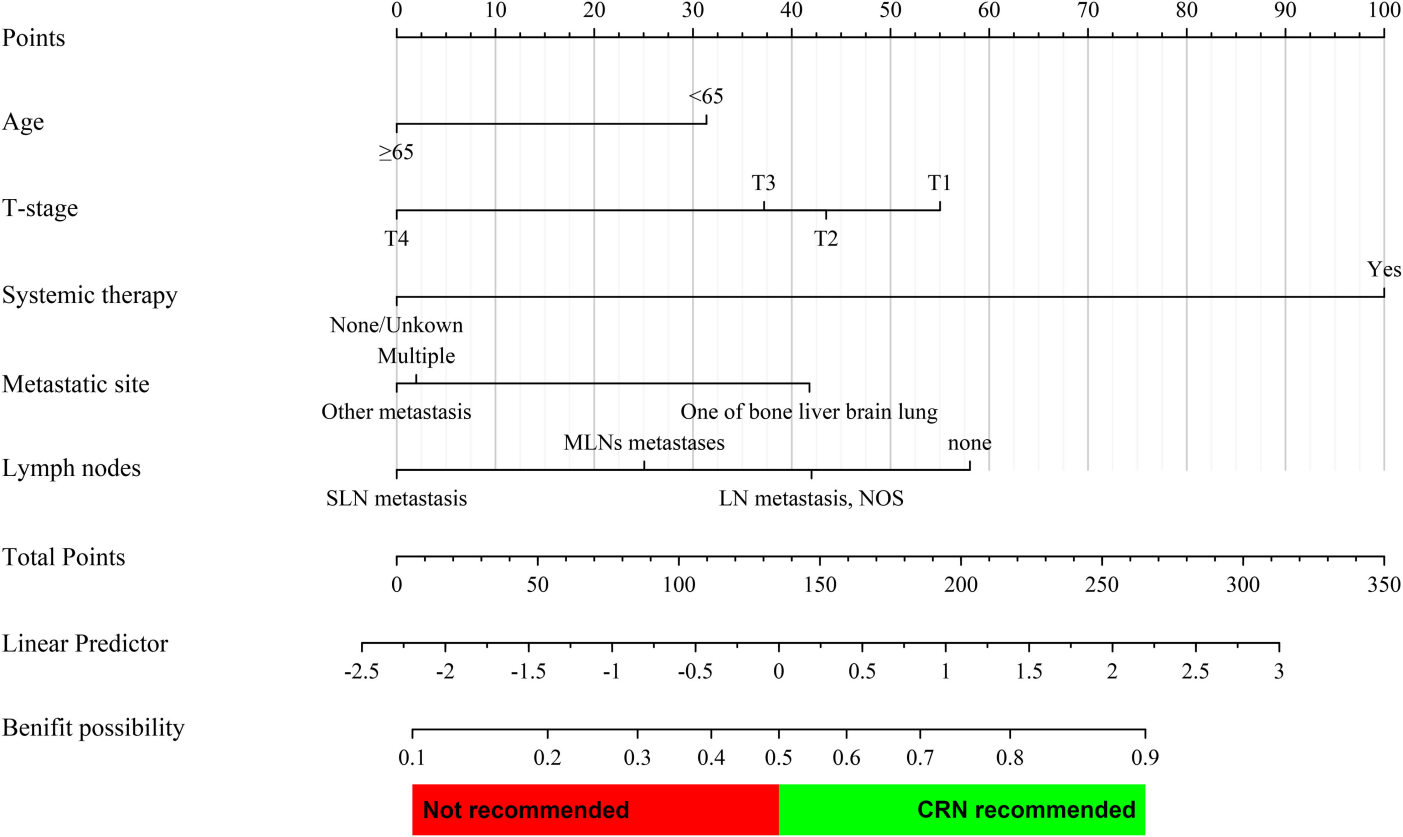
Prediction nomogram for assessing the probability that a patient with metastatic sarcomatoid renal cell carcinoma may benefit from CRN. The probability of each variable was converted into scores and summed to obtain the total score. The cutoff point of the nomogram was 0.5, and a patient was assumed to benefit from CRN if the total prediction probability was >0.5.

According to the multivariate Cox regression analysis of the CRN group (*n* = 608) before PSM, clinical variables that could be accessed preoperatively were included for constructing the nomogram, including age, T-stage, systematic therapy, metastatic site, and lymph nodes. Based on multivariate logistic regression analysis, a prediction model was established as a nomogram to predict the probability that a patient with sarcomatoid mRCC may benefit from CRN (**Figure 4**).

**Figure 5:**
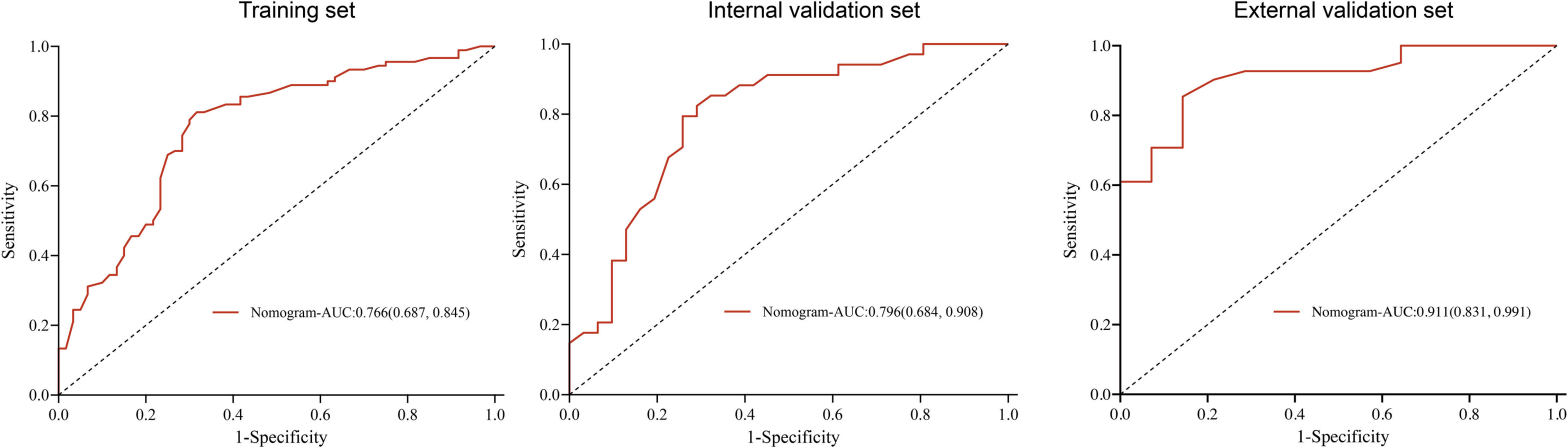
Receiver operating characteristic curve of the nomogram in the training, internal validation, and external validation sets.

**Figure 6:**
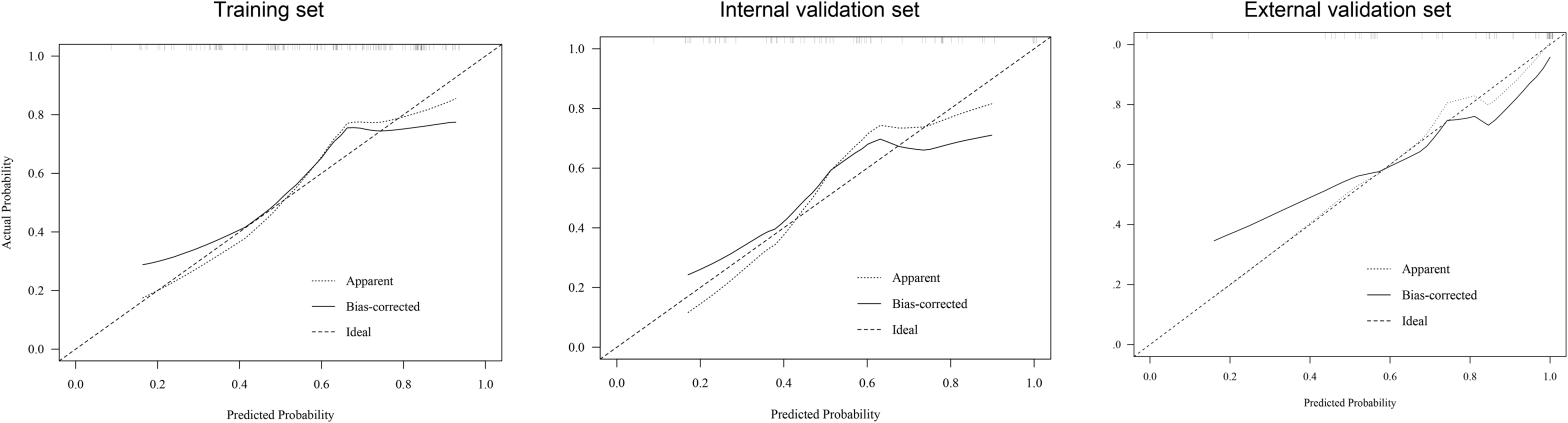
Calibration curve of the nomogram in the training, internal validation, and external validation sets.

The prediction nomogram showed a good discrimination capacity for identifying patients with mRCC who may benefit from CRN in the training cohort (AUC = 0.766, 95% CI: 0.687–0.845), internal validation cohort (AUC = 0.796, 95% CI: 0.684–0.908), and external validation cohort (AUC = 0.911, 95% CI: 0.831–0.991) (**Figure 5**). The outcomes of the actual calibration curve for training, internal validation, and external validation cohorts were all in perfect agreement with those of the nomogram (**Figure 6**).

### 3.4 Clinical utility of the prediction nomogram

All corresponding scores of each variable were superimposed in the nomogram to calculate the CRN benefit probability. According to the total score from the nomogram, candidates with a predicted probability >0.5 at the cutoff point were stratified as the CRN benefit group. Otherwise, they are stratified as the no CRN benefit group.

**Figure 7:**
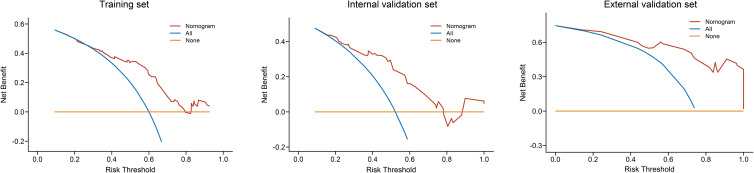
Decision curve analysis of the nomogram in the training, internal validation, and external validation sets.

**Figure 8:**
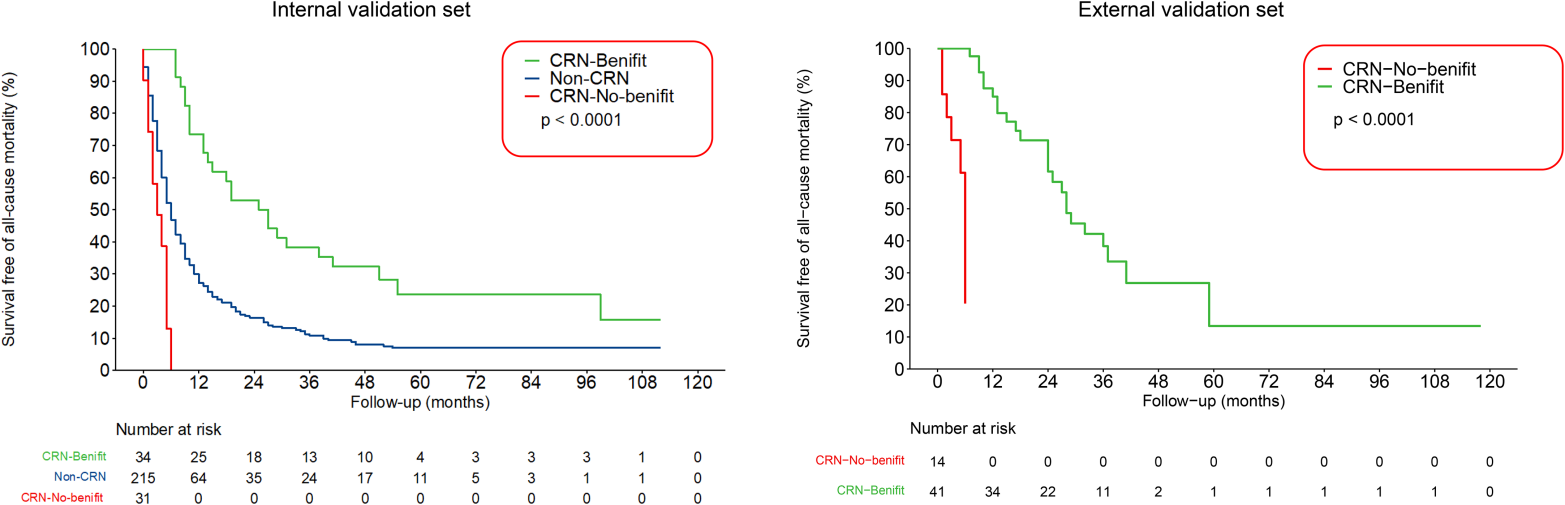
Kaplan–Meier survival curves of patients with metastatic sarcomatoid renal cell carcinoma classified based on the nomogram as CRN benefit and no CRN benefit groups in the internal and external validation sets.

Decision curve analysis showed the clinical value of the nomogram (**Figure 7**). The Kaplan–Meier analysis showed that the OS of the CRN benefit group, no CRN benefit group, and non-CRN group was accurately distinguished in both the internal and external validation sets, suggesting good clinical value of the nomogram (**Figure 8**).

## 4 Discussion

Sarcomatoid RCC is a unique pathological feature that is observed in approximately 5% of all RCC cases and 20% of all metastatic RCC cases (13). Genomic and transcriptomic sequencing of sarcomatoid clear cell RCC revealed similar molecular omics characteristics between epithelioid and sarcomatoid components (14); another study reported that these components share the same clonal origin (15). However, they are completely different from the nonsarcomatoid components of RCC (16, 17). Thus, we cannot simply treat sarcomatoid RCC as a unique pathological feature but as a special type of RCC that is more aggressive and is associated with a poorer prognosis.

Cytoreductive nephrectomy, particularly CRN, for metastatic RCC has evolved considerably over the past decade. However, whether and how many patients with sarcomatoid mRCC benefit from CRN remains unknown. Shuch et al. compared the results of cytoreductive nephrectomy in patients with metastatic RCC with or without sarcomatoid features and revealed that those with sarcomatoid mRCC undergoing cytoreductive nephrectomy had a poor prognosis with a moderate OS (18). Several studies on sarcomatoid mRCC presented similar conclusions (19, 20). Therefore, it is necessary to select appropriate patients for CRN.

In this study, we compared OS and CSS between different subgroups of patients with sarcomatoid mRCC treated with or without CRN after balancing other variables that affect OS. This study showed that most patients who underwent CRN lived longer than those without CRN in the matched groups; however, not all of them had a longer survival time than those who did not receive CRN. Thus, we should make full use of the effectiveness of CRN, which further demonstrates the importance of our research.

Sarcomatoid RCC has higher expression levels of PD-1 and PD-L1 and tumor-associated lymphocyte infiltration than common pathological types of RCC (21–23). Several clinical trials have shown that immunotherapy can achieve higher objective response rates and longer survival in sarcomatoid RCC than any previous treatment modality (24–26). Accordingly, systematic therapy is heavily weighted in our prediction model.

Interestingly, our nomogram showed that, compared with multiple-organ metastases, metastases other than the bone, liver, brain, and lung had similar effects on patients with CRN. Meanwhile, individuals with bone, liver, brain, or lung metastasis appear to be more likely to benefit from CRN. This is because the tumor burden of a single bone, liver, brain, or lung metastasis is lower than that of multiple or even other metastatic organs. More studies are needed because of the lack of detailed information on other metastatic organs.

In the multivariable Cox regression, “N stage” was not selected as one of the variables in our nomogram. Instead, “lymph nodes,” which reveal more specific details of the lymph node metastases, were considered in our prediction model. To our surprise, patients with sarcomatoid mRCC having multiple regional lymph node metastases benefited more from CRN than those with a single regional metastasis. This may be due to the lymph node dissection performed during CRN. Increasing lymph node yield on the pathologic specimen is associated with improved OS in patients with mRCC and N1 disease who underwent cytoreductive nephrectomy (27). Although lymph node dissection did not improve OS in mRCC in many studies, the N stage should not be simply divided into N0 and N1 as in the current TNM stage, which is in line with the new EAU RCC TNM staging system (28).

Despite the high accuracy of our prediction model in selecting patients suitable for CRN, this study has several limitations. First, prognostic variables such as patient general conditions, surgical complications, laboratory tests, and pathological descriptions are missing in the SEER database. Second, the SEER database lacks information about the percentage of the sarcomatoid tumor components, which might have affected the treatment outcomes. Third, the Furhman grade was not considered a variable because only 9.2% of sarcomatoid mRCC cases could be identified based on preoperative biopsy (29). In addition, no detailed information regarding systemic therapy was available for this cohort of patients with sarcomatoid mRCC. Thus, it remained unclear whether systemic therapy was balanced between CRN and non-CRN groups. As we retrieved data from patients with sarcomatoid mRCC diagnosed between 2010 and 2015, it was presumed that most of them received standard systematic therapy based on the American Urological Association guideline recommendations. Fourth, all studies using the SEER database are limited by their retrospective nature. Finally, the lack of randomization in our study indicates the possibility of differences between CRN and non-CRN groups.

To the best of our knowledge, this is the first study to establish a prediction model to select patients with sarcomatoid mRCC who are suitable for CRN. Future studies should focus on external validation with a large sample size and a prospective randomized controlled design.

CRN can improve survival in patients with sarcomatoid mRCC. We successfully constructed a nomogram with good accuracy to select patients suitable for CRN. This tool may provide more precise treatment strategies for patients with sarcomatoid mRCC.

## Data availability statement

The original contributions presented in the study are included in the article/supplementary material. Further inquiries can be directed to the corresponding authors.

## Ethics statement

The studies involving humans were approved by ethics committee of Nanjing University Jinling Hospital, Affiliated Hospital of Medical School, Nanjing University; ethics committee of Changhai Hospital, Naval Medical University; and ethics committee of Qilu Hospital of Shandong University. The studies were conducted in accordance with the local legislation and institutional requirements. The participants provided their written informed consent to participate in this study.

## Author contributions

YZ contributed to the study design and drafting of the manuscript. CH, JG, and LQ contributed to the editing and reviewing of the manuscript. CT contributed to data collection. YG and XZ contributed to the statistical analysis. JD, SX, and ZS contributed to the operation of the study. JH, TS, and HH contributed to the validation. XY and WZ contributed to the supervision of the study. All authors contributed to the article and approved the submitted version.
